# Strategies for extremity reconstruction with exposed bones and tendons using acellular dermal matrices: concept of sequential vascularization

**DOI:** 10.1080/23320885.2021.2011289

**Published:** 2021-12-28

**Authors:** Ajul Shah, Philippe Taupin

**Affiliations:** a The Plastic Surgery Center, Institute for Advanced Reconstruction, Shrewsbury, NJ, USA; b Medical Affairs, Integra LifeSciences, Princeton, NJ, USA

**Keywords:** Acellular dermal matrices, exposed bone and tendon, vascularization, wound reconstruction

## Abstract

We report 3 cases of patients treated with Bilayer Wound Matrix over exposed structures. In all patients, dermal matrices revascularization occurred sequentially over the course of 6–12 weeks, leading to successful wound closure. Acellular dermal matrices allow more difficult areas with poor vascularity to be covered from the ‘inside-out’.

## Introduction

Successful take of skin grafts requires a well vascularized wound bed and remains a challenge for plastic surgeons particularly when performed over poorly vascularized structures, such as exposed bones and tendons. Using the guiding principles of the reconstructive ladder, trauma and plastic surgeons have different options to choose from when facing wound reconstruction [1]. Local and regional flaps cover bony surfaces and tendons, but are limited by the size of the defect [2]. Pedicled and free flaps allow coverage of large tissue defects, but are associated with substantial donor-site morbidity [3]. Advanced technologies, such as dermal matrices, are now integral part of the reconstruction ladder/elevator, adding new opportunities for vascular ingrowth and subsequent graft take [4].

Integra^®^ Dermal Regeneration Template (IDRT, Integra LifeSciences, Princeton NJ, USA) and Integra^®^ Bilayer Wound Matrix (IBWM, Integra LifeSciences, Princeton NJ, USA) are xenogenic acellular dermal matrices (ADMs) [5]. ADMs are a class of biological, synthetic, and composite scaffold materials used to augment and replace deficient or missing skin and soft tissues [6]. IDRT and IBWM are bilayer matrices composed of a layer of cross-linked bovine tendon collagen and glycosaminoglycans (chondroitin-6-sulfate-derived from shark cartilage), and a silicone layer [7]. The collagen layer provides structure and stability, and enables infiltration of macrophages, fibroblasts, lymphocytes and endothelial cells, resulting in the generation of a new vascular network and the formation of a neodermis. The temporary silicone layer, by simulating the physiological function of the epidermis, modulates fluid flux and acts as a mechanical barrier to bacterial invasion [8]. Both matrices are applied to the wound in a 2-stage procedure [5]. First, the wound is excised and debrided, and the dermal matrix is placed over the bed wound. Second, after 3–4 weeks, when neovascularization is achieved and the neodermis is being formed, the silicone layer is removed and replaced by a split-thickness thin graft (STSG) [9]. Over the years, IDRT and IBWM have been used to treat a broad range of wounds from burns, acute and chronic wounds to cancer resection and scar reconstruction [10–15]. Meshed variants of IDRT and IBWM, Integra^®^ Meshed Dermal Regeneration Template (IMDRT), and Integra^®^ Meshed Bilayer Wound Matrix (IMBWM) respectively, are available and may be used in conjunction with vacuum-assisted closure (VAC) therapy.

IDRT and IBWM have been used to treat a broad range of wounds, particularly over challenging wounds such as with exposed bone and tendon [16–19]. In contrast, exposed bone, tendon, or cartilage do not have sufficient vascularity to support a granulation bed for re-epithelialization or neovascularization for skin graft survival [20]. In this mansucript, we report 3 cases of adult patients with challenging wounds managed using IMBWM over exposed structures. By providing sequential vascularization, IMBWM allows more difficult areas with poor vascularity to be covered from the ‘inside-out’. The bilayer matrix, in contrast to skin graft, is not ‘time sensitive’ and do not have the same chance as ‘dying’ – it can instead incorporate sequentially over time, providing a healed reconstruction in situations where skin grafts could not.

## Case reports

The study was conducted following the principles outlined in the Declaration of Helsinki. All patients were informed of the pros and cons of IMBWM treatment, and signed a consent approving or rejecting such information. All patients gave informed written consent for the use of the data collected.

### Case 1

**Figure 1.**
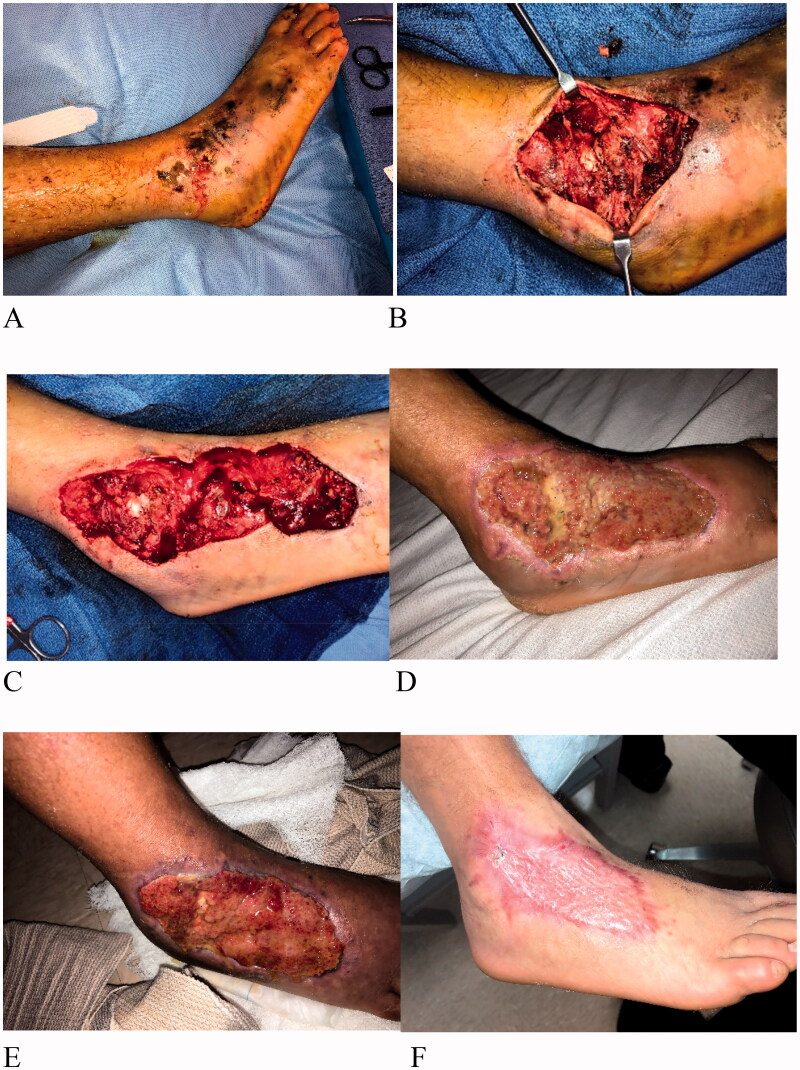
Case 1. A 34-year-old male while riding a bicycle was hit by motorcycle. He suffered soft tissue injury on the right foot. The wound on the foot (A) had exposed bone and joint (B) and was debrided, prior to matrix placement (C). A sheet of IMBWM was applied to the wound. Vascularization of the dermal matrix occurred sequentially (D) and was complete 9 weeks after matrix placement (E), at which time the silicon layer was removed and STSG was applied. STSG take 100% was observed after 1 week (F).

A 34-year-old male was hit by a motorcycle while riding a bicycle. He was admitted to the hospital and presented a soft tissue injury on the right foot; on the dorsal side lateral to the ankle with exposed bone and joint (Figure 1(A,B)). Patient was a smoker with no medical history. The wound was contaminated with significant amount of gravel and road rash. The patient was administered antibiotics upon admission, and the wound underwent 2 operative debridement procedures (Figure 1(C)). Following debridement, the resulting defects measured 15 × 8 cm with exposed underlying joint. IMBWM was applied to the wound in the operating room (OR) under general anesthesia, and was fixed in place using staples. The immediate post-operative dressing was a wound VAC, with black foam sponge, set at negative pressure of 125 mm Hg. The patient was discharged 2 days after placement of the matrix, with the outpatient wound VAC. Vascularization of the dermal matrix occurred sequentially (Figure 1(D)) and was achieved 9 weeks after placement (Figure 1(E)), at which time the silicone layer was removed. Matrix take was 100%. A STSG of 12/1000″ was applied to the neodermis, under general anesthesia. The patient was discharged the same day after grafting, with 5 days of outpatient wound VAC treatment. STSG take was 100%, 1 week after grafting (Figure 1(F)). There was no postoperative complication, and the reconstruction covered the exposed joint without flap treatment.

### Case 2

**Figure 2.**
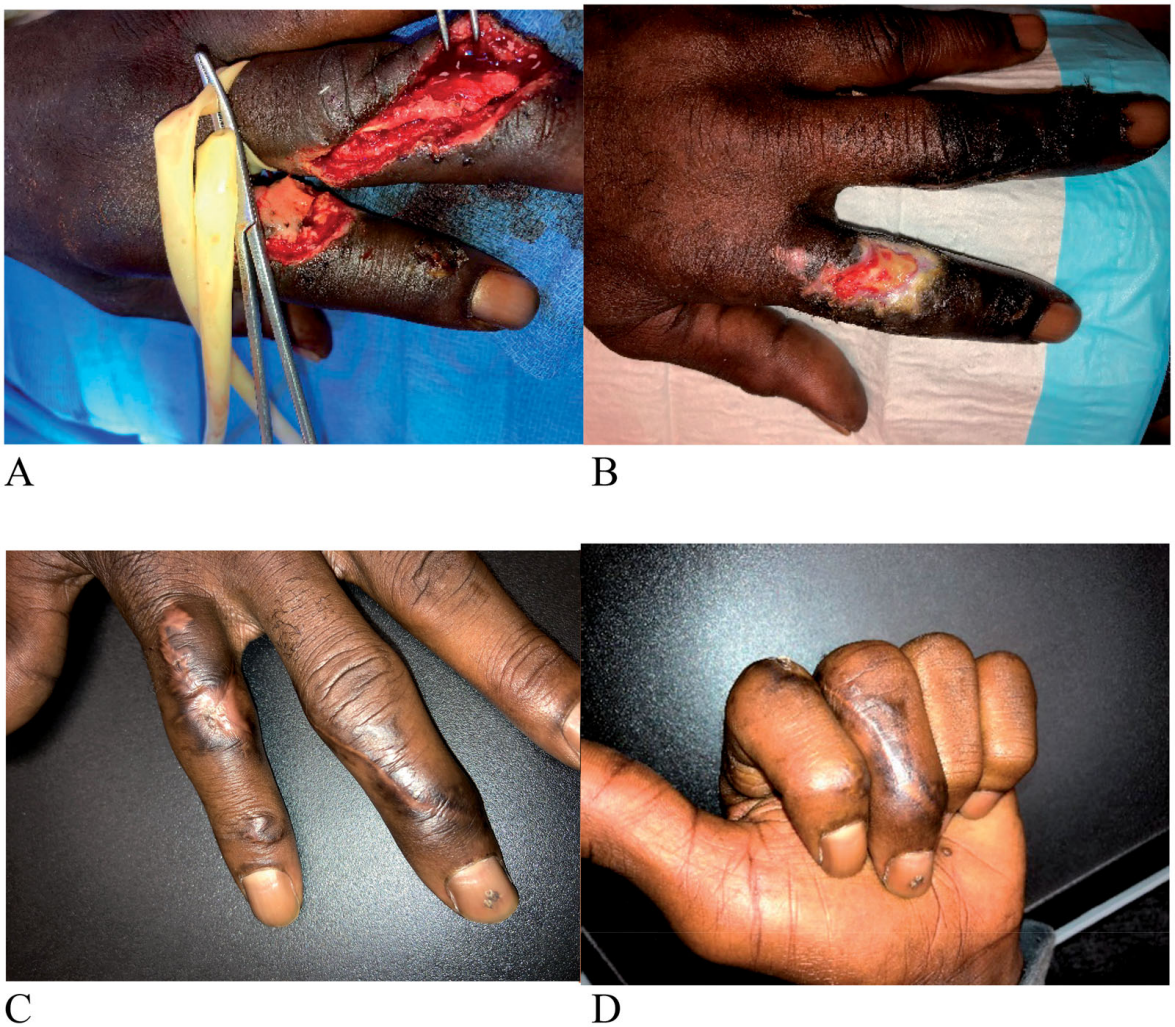
Case 2. A 65-year-old male admitted to the hospital for injuries to his dorsal 2^nd^ and 3^rd^ fingers, after a table saw injury (A). The injury to the left index finger was inclusive of a unicortical fracture of the proximal phalanx with an absence of periosteum. After debridement of the dorsal 2nd finger, a sheet of IMBWM was applied to the wound. Vascularization of the dermal matrix on the dorsal 2^nd^ finger occurred sequentially (B and C) and continued 3 weeks after placement, at which time the silicone layer was removed. To allow for time for sequential vascularization of the IMBWM to occur, the wound was subsequently allowed to heal by secondary intention. Patient recovered full ROM on his 2^nd^ finger with a fully healed reconstruction, after 12 weeks of treatment (D).

A 65-year-old male was admitted to the hospital with injuries to his left arm dorsal 2^nd^ and 3^rd^ fingers with a table saw (Figure 2(A)). Patient had a history of uncontrolled hypertension, diabetes, and was a heavy smoker. The patient was administered intravenous (IV) antibiotics. The injury to the left index finger was inclusive of a unicortical fracture of the proximal phalanx with an absence of periosteum. The wound on the dorsal 2^nd^ finger underwent 1 operative debridement procedure, which left a defect measuring 3 × 2 cm with exposed bone. IMBWM was applied to the wound and fixed in place using a running absorbable suture. Post-operative dressing of the wound included bacitracin, adaptic and a small gauze wrap. The patient was discharged the same day after placement of the matrix. Vascularization of the dermal matrix occurred sequentially (Figure 2(B,C)) and continued 3 weeks after placement, at which time the silicone layer was removed. Matrix take was approximately 60% at that time. IMBWM was applied with the aim to perform two-stage procedures. However, due to the desire to allow for time for sequential vascularization of the IMBWM to occur, the wound was subsequently allowed to heal by secondary intention. Patient recovered full ROM on his 2^nd^ finger with a fully healed reconstruction after 12 weeks of treatment (Figure 2(D)).

### Case 3

**Figure 3.**
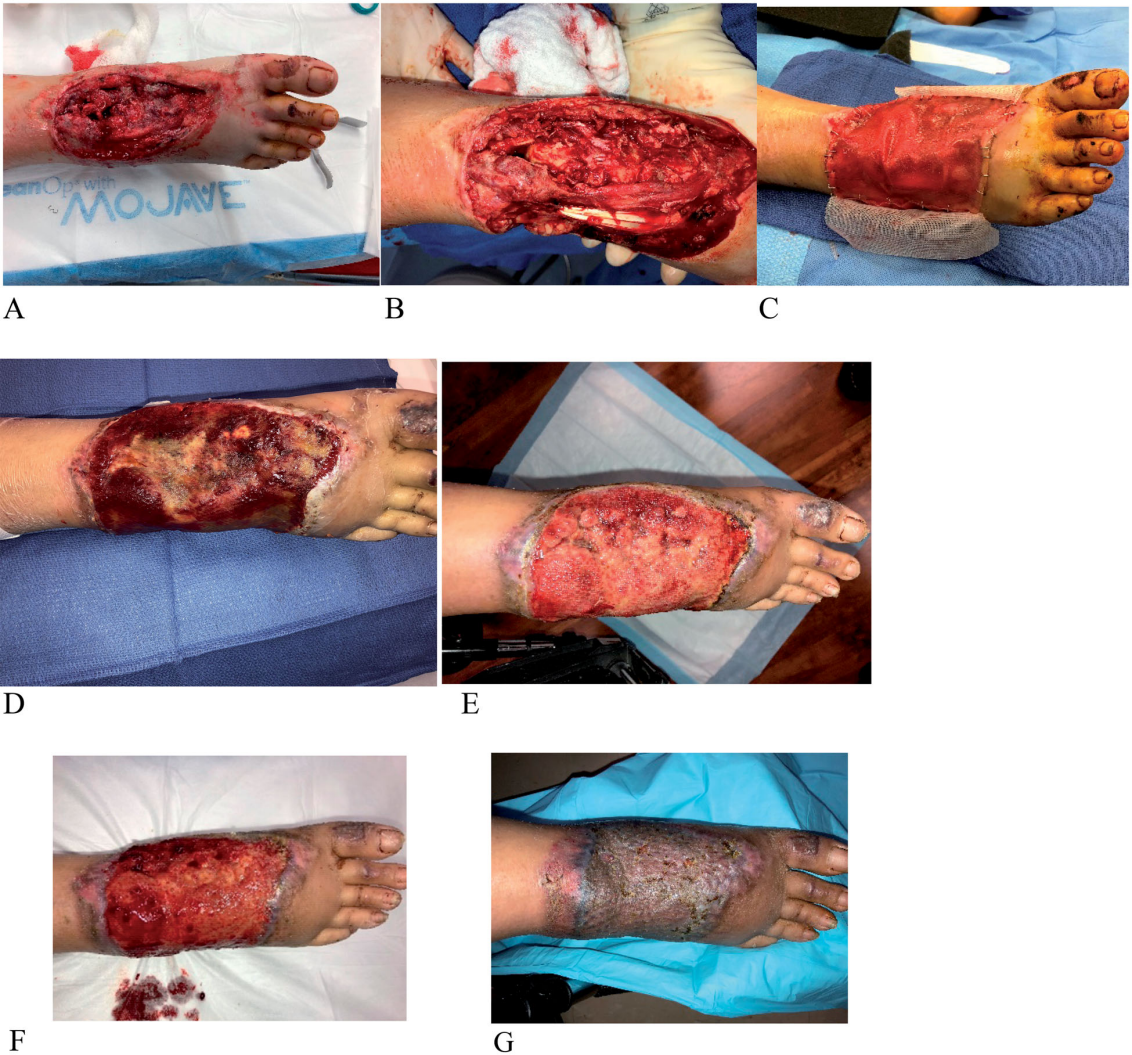
Case 3. A 48-year-old female admitted to the hospital after a scooter crash. She suffered a lower extremity right dorsal foot wound with exposed bone and tendons. The wound was debrided twice prior to matrices placement (A, B). IWM Thin was stacked over the wound bed to allow for rapid incorporation over the exposed vital structures, and a sheet of IMBWM was applied over the wound (C). Vascularization of the matrices occurred sequentially after matrices placement; partial vascularization at 3 weeks (D), improved vascularization at 5 weeks (E). The vascularization was robust at 6 weeks (F), at which time the silicon layer was removed and STSG was applied. The wound was completely healed and showed good cosmesis 3 months after skin grafting (G).

A 48-year-old female was riding on the back of a scooter when she was involved in a crash and was thrown from the scooter. She was admitted to the hospital and presented a lower extremity trauma; a wound dorsal on her right foot with exposed bone and tendons. The wound was contaminated with gravel and dirt. Patient had Moyamoya disease, mental disabilities, was a non-smoker and was cared for by family. Though the patient was a medical candidate for a free flap procedure, due to concerns of the patient not being able to tolerate a large-scale intervention and being unable to follow post-operative directions, it was decided to treat the patients using an ADM instead. The patient was unable to follow directions while in the hospital, and it was determined that leg elevation that is mandatory after free flap treatment would not be able to be followed for an extended period of time. Patient underwent 2 operative debridement procedures (Figure 3(A,B)), after being administered IV antibiotics. Following debridement, the resulting defects measured 10 × 12 cm. Integra Wound Matrix Thin (IWM Thin, Integra LifeSciences, Princeton NJ, USA) was applied to the wound bed to obtain enough volume for reconstruction, to allow for rapid incorporation over the exposed vital structures. During the same surgical procedure, a sheet of IMBWM was stacked with IWM Thin over the wound bed, and fixed in place using staples in the OR under general anesthesia (Figure 3(C)). Post-operative wound VAC therapy was instituted, using black foam, set at 125 mm Hg. The patient was discharged the same day after placement of the matrices. Vascularization of the dermal matrices occurred sequentially (Figure 3(D,E) and was achieved 6 weeks after placement (Figure 3(F)), at which time the silicone layer was removed. Although the matrix had not vascularized completely by 3 weeks, this was not a failure in treatment. By 6 weeks, the matrix vascularization was 100%. A STSG (10/1000″) was applied to the neodermis, under general anesthesia. The patient was discharged the same day after grafting, with 5 days of outpatient wound VAC treatment. STSG take was 100%, after 1 week. There was no post-operative complication, and the patient healed the severe wound without flap reconstruction. The wound was completely healed and showed good cosmesis 3 months after skin grafting (Figure 3(G)).

## Discussion

We report a case series of 3 adult patients treated with IMBWM for soft tissue defects of the extremity with exposed bones and tendons. In all 3 patients, revascularization of the dermal matrices occurred sequentially over the course of 6–12 weeks. While Patients 1 and 3 underwent a 2-stage procedure with STSG, Patient 2 underwent single-stage re-epithelialization due to patient preference and the desire to allow for time for sequential vascularization of the ADM to occur. All 3 patients showed satisfactory cosmesis and recovered full ROM after surgery.

The 3 patients (age range 34-65-year-old) reported had co-morbidities, i.e. smoker, uncontrolled hypertension, diabetes (Patients 1 and 2), while one patient had Moyamoya disease and mental disabilities (Patient 3), which did not affect the successful surgical procedures. In the 3 patients treated using IMBWM, the dermal matrices and the subsequent skin grafts (Patients 1 and 3) integrated successfully in the various extremity wound types treated, i.e. soft tissue injuries on the foot and fingers, with exposed bone and tendons. In all, for these 3 patients, the integration of the matrices in the wound beds was not affected by the patients’ profiles, i.e. co-morbidities, wound etiology, previous infection, anatomy and wound characteristics.

Four distinct phases of dermal regeneration have been observed in histological studies in patients requiring reconstructive surgery for contracture release using IDRT, i.e. imbibition, fibroblast migration, neovascularization, and remodeling and maturation [9]. Full vascularization of the neodermis occurred at 4 weeks, at which point the silicone membrane is replaced with a STSG. The second-stage of wound reconstruction using the bilayer matrix is largely dependent on the state of the granulation bed, and the average period between the matrix application and STSG have been reported over the course of 3–6 weeks. The process of vascularization of the bilayer matrices can be appreciated clinically by the color progression from pink to pale yellow to peach [9]. In our study, the periods between the matrix application and full vascularization of the neodermis were 9, 12 and 6 weeks for patients 1, 2 and 3, respectively, with the color progression from pink to pale yellow to peach observed in each case, as described by Moiemen et al. [9]. Literature evidence shows IDRT and IBWM provide high quality coverage over wounds of various etiologies with exposed bone or tendon where it is efficient in stimulating the creation of a new vascularized dermis [16–19]. The lengthier process of vascularization of the matrix, observed in Patients 1,2 and 3, with IMBWM for soft tissue defects of the extremity, with exposed bones and tendons, reflects a process of sequential vascularization necessary for the successful dermal regeneration of soft tissue in those wounds. VAC therapy, a mean of applying subatmospheric pressure to a wound, has been reported to contribute to the healing process *via* several mechanisms, e.g. removal of exudate, stimulation of local growth factors, angiogenesis, and granulation tissue formation [21–23]. It may have contributed to a more rapid incorporation times with improvements in healing rates in cases 2 and 3. Due to the limited number of cases − 3 cases - reported in the study, we noticed neither any relationships between patients comorbidities and time to vascularization of the dermal matrix, nor any other factor that could influence the process. Skin graft, though a widely used technique, is not able to cover bone prominences or cavities. Our results showed that by providing sequential vascularization, IMBWM led to successful wound closure in all 3 patients. The ADM is not ‘time sensitive’ and does not have the same chance as ‘dying’, as does skin graft.

IDRT and IBWM have been used to treat a broad range of wounds, particularly over challenging wounds such as with exposed bone and tendon, where the healed wound with exposed structures were reported as very pliable and uniform in color and texture, and not adhering to the deeper structures, providing a gliding plane for tendons particularly [16,24,25]. This latter aspect is important as it allows for complete ROM when the bilayer matrices are applied over joints, tendons, or movable structures. In contrast, skin grafts and many flaps result in a high rate of tendon adhesion [26]. In the 3 patients treated, the use IMBWM led to the successful re-epithelialization and reconstruction of the skin with all patients recovering full ROMs, as well.

We report 3 cases of patients undergoing soft tissue reconstruction using IMBWM; 2 patients undergoing a 2-stage procedure with STSG, and 1 patient undergoing a single-stage procedure with re-epithelialization by secondary intention (Patient 2). Integra bilayer matrices, IDRT and IBWM, are applied to the wound in a staged-procedure, requiring at least two surgical operations: a first one for application of the matrix and a second one to remove the silicone layer with placement of a subsequent STSG. It has been reported that staged-procedures using IDRT should be used when possible, as it provides effective and durable results for all defect sizes, patients with relatively small wound. Those with more superficial wounds, particularly if other co-morbidities that would make repeated intubation and anesthesia more risky, might benefit from a single-stage procedure with re-epithelialization by secondary intention [27]. One can hypothesize that in smaller wounds, so much contraction has occurred once the silicone is removed, that there is little need for skin graft. In the 3 cases we reported, the decision to use a staged-procedure or not for reconstruction was made on a case-by-case basis due to the mature of the wounds and the process for sequential vascularization. Hence for Patient 2, the choice to use a single-stage procedure with re-epithelialization by secondary intention of soft tissue reconstruction was made due to patient preference and the desire to allow for time for sequential vascularization.

Stacking dermal matrices is a mean to obtain enough volume for reconstruction. Carothers et al. [28] reported on the use of bilayer matrices for reconstruction of a soft tissue defect after tumor excision from the palm of the hand. In their case report, 2 sheets of bilayer matrices were stacked directly over a wound bed with exposed flexor tendons and median nerve. The authors reported full functional use of the hand with mild paresthesias and no adhesions of the flexor tendons underlying the ADMs [28]. The stacking of IBWMs serially has been reported to address defect over the palm of the hand in terms of depth, leading to excellent wound healing and coverage and full ROM [27]. In Patient 3, IWM Thin was stacked concomitantly with IMBWM to the wound bed to obtain enough volume for reconstruction, to allow for rapid incorporation over the exposed vital structures, and consistent with the generation of healed wound not adhering to the deeper structures, providing a gliding plane for tendons particularly, as well. In very deep tissue loss or in certain situations in which cosmesis is important, a second or successive layers of a dermal matrix might be very worthwhile [27]. However, there are complications of stacking dermal matrices reported in the literature. Phenomenon of ‘ghosting’, epithelial autografts applied to the matured neodermis auto-digest to dispersed cells followed subsequently by the reappearance of a confluent epithelial layer, has been reported on multilayer IDRT applications. Whether this is a manifestation of the (hypo) vascularity of the multiple layer approach and/or the vagaries of local implementation of the IDRT technique remained unclear [27]. In addition, any potential benefit achieved through stacking of dermal matrices would have to be weighed against the costs required for multiple layer of products and/or surgical procedures. The use of multiple layers of IDRT should be seen as an unusual circumstance for cases of very deep tissue loss, and not the norm [27].

IDRT and IBWM are reported to survive well in vascularized areas where the neodermis can get good vascularity from the wound bed and surrounding tissue. When apposed straight to the bone, the process of capillaries invasion of the dermal matrices came from the bed and from the margins of the wound; without periosteum the healing process came only from the margins of the wound [29]. The vascular channels were restored from underneath the paratenon and surrounding tissues. It allows for neovascularized tissue to form over exposed or denuded structures within these wounds, creating a more robust vascularized tissue bed for neovascularization of subsequently placed skin grafts than skin grafts and many flaps [20,30]. Our results showed that if there is a good area of vascularity, the ADM will continue to vascularize from the ‘inside out’ over a period-of-time. IMBWM allows more difficult areas with poor vascularity to be covered, by sequential vascularization. The same cannot be said of skin grafts, which are time sensitive in terms of their incorporation and will die within a short period of time without vascularity.

There are limitations, contra-indications and advantages of using dermal matrices for wound reconstruction. Among them, not all wound injuries justify the use of dermal matrices and dermal matrices may not represent the best option for all deep soft-tissue injuries. The use of dermal matrices has an initial steep learning curve, which can be complicated by infection, hematoma and seroma formation. IDRT and IBWM should not be used in patients with known sensitivity to bovine collagen or chondroitin materials. The cost of the dermal matrix to be applied and the insurance coverage of such therapies remain challenges, and must be balanced against the associated costs of an alternate procedure, such as flap coverage, and patient’s outcomes.

## Conclusion

ADMs are integral part of the reconstructive armamentarium. The use of ADMs in extremity reconstruction often involves two-stage reconstruction. Results from these 3 cases highlight the power of dermal matrices for treating challenging wounds with exposed vital structures. By providing sequential vascularization, IMBWM allows more difficult areas with poor vascularity to be covered from the ‘inside-out’. The bilayer matrix, in contrast to skin graft, is not ‘time sensitive’ and do not have the same chance as ‘dying’.
